# Constructing High-Fidelity Phenotype Knowledge Graphs for Infectious Diseases With a Fine-Grained Semantic Information Model: Development and Usability Study

**DOI:** 10.2196/26892

**Published:** 2021-06-15

**Authors:** Lizong Deng, Luming Chen, Tao Yang, Mi Liu, Shicheng Li, Taijiao Jiang

**Affiliations:** 1 Center of Systems Medicine Institute of Basic Medical Sciences Chinese Academy of Medical Sciences & Peking Union Medical College Beijing China; 2 Suzhou Institute of Systems Medicine Suzhou China; 3 Jiangsu Institute of Clinical Immunology Jiangsu Key Laboratory of Clinical Immunology The First Affiliated Hospital of Soochow University Suzhou China; 4 Guangzhou Laboratory Guangzhou China

**Keywords:** knowledge graph, knowledge granularity, machine learning, high-fidelity phenotyping, phenotyping, phenotype, semantic

## Abstract

**Background:**

Phenotypes characterize the clinical manifestations of diseases and provide important information for diagnosis. Therefore, the construction of phenotype knowledge graphs for diseases is valuable to the development of artificial intelligence in medicine. However, phenotype knowledge graphs in current knowledge bases such as WikiData and DBpedia are coarse-grained knowledge graphs because they only consider the core concepts of phenotypes while neglecting the details (attributes) associated with these phenotypes.

**Objective:**

To characterize the details of disease phenotypes for clinical guidelines, we proposed a fine-grained semantic information model named PhenoSSU (semantic structured unit of phenotypes).

**Methods:**

PhenoSSU is an “entity-attribute-value” model by its very nature, and it aims to capture the full semantic information underlying phenotype descriptions with a series of attributes and values. A total of 193 clinical guidelines for infectious diseases from Wikipedia were selected as the study corpus, and 12 attributes from SNOMED-CT were introduced into the PhenoSSU model based on the co-occurrences of phenotype concepts and attribute values. The expressive power of the PhenoSSU model was evaluated by analyzing whether PhenoSSU instances could capture the full semantics underlying the descriptions of the corresponding phenotypes. To automatically construct fine-grained phenotype knowledge graphs, a hybrid strategy that first recognized phenotype concepts with the MetaMap tool and then predicted the attribute values of phenotypes with machine learning classifiers was developed.

**Results:**

Fine-grained phenotype knowledge graphs of 193 infectious diseases were manually constructed with the BRAT annotation tool. A total of 4020 PhenoSSU instances were annotated in these knowledge graphs, and 3757 of them (89.5%) were found to be able to capture the full semantics underlying the descriptions of the corresponding phenotypes listed in clinical guidelines. By comparison, other information models, such as the clinical element model and the HL7 fast health care interoperability resource model, could only capture the full semantics underlying 48.4% (2034/4020) and 21.8% (914/4020) of the descriptions of phenotypes listed in clinical guidelines, respectively. The hybrid strategy achieved an F1-score of 0.732 for the subtask of phenotype concept recognition and an average weighted accuracy of 0.776 for the subtask of attribute value prediction.

**Conclusions:**

PhenoSSU is an effective information model for the precise representation of phenotype knowledge for clinical guidelines, and machine learning can be used to improve the efficiency of constructing PhenoSSU-based knowledge graphs. Our work will potentially shift the focus of medical knowledge engineering from a coarse-grained level to a more fine-grained level.

## Introduction

When people are sick, their bodies present a series of observable or perceptible abnormalities, which are called phenotypes. In medicine, the phenotype concept covers signs and symptoms, laboratory test results, and imaging findings [1]. Phenotypes characterize the clinical manifestations of diseases, which provide important clues for diagnoses. Knowledge about disease phenotypes is usually documented as free text in medical textbooks or clinical guidelines, and such knowledge forms are hard for computers to use. Therefore, it is essential to transform phenotype knowledge into a machine-understandable format to facilitate the development of automated systems that could improve health care [2].

To date, many structured knowledge bases, such as WikiData [3], MalaCards [4], and DBpedia [5], have been constructed for disease phenotypes. In these knowledge bases, the phenotype knowledge of a disease is represented as a list of phenotype concepts or terms (Multimedia Appendix 1). However, such a concept-based representation only focuses on the presence or absence of a phenotype but neglects its contextual properties [6,7]. The description “sudden, severe abdominal pain in the lower right abdomen,” for example, names three attributes of abdominal pain, including the onset pattern (sudden), severity (severe), and quadrant pattern (lower right abdomen). These attributes are valuable for diagnosis but missing in the provided concept-based representation. Due to the neglect of phenotypic details, current phenotype knowledge bases only characterize disease manifestations at a very coarse-grained level [8], which is considered to be “sloppy and imprecise” [9,10].

To precisely represent phenotype knowledge in clinical guidelines, it is necessary to introduce fine-grained semantic information models [11], which consider phenotypes and attributes simultaneously. The currently available semantic models for representing phenotype information include but are not limited to clinical element models (CEMs) [12], the Health Level Seven fast health care interoperability resource (FHIR) model [13], and the clinical quality language model [14]. All these models can be viewed as standard entity-attribute-value structures, which represent phenotype information with sufficient details by using various attributes and qualifier values. For example, a CEM model considers 17 attributes associated with phenotypes, such as phenotype severity, laterality, and duration. Although semantic information models such as CEM and FHIR have defined many attributes for phenotypes, it should be noted that these attributes are mainly designed for recording phenotypic information in electronic medical records. However, the logic underlying phenotypic descriptions in clinical guidelines is quite different from that in electronic medical records. For example, clinical guidelines usually describe the frequency of a phenotype in a population (eg, 30% of patients may have a loss of smell); however, this attribute is not defined in CEM and FHIR models. In addition to the frequencies of phenotypes, other often used attributes such as temporal patterns (eg, acute, chronic) and pain characteristics (eg, sharp, dull) are also neglected by the CEM and FHIR models. Therefore, it is necessary to optimize the attributes included in current information models to make them more suitable for representing phenotype knowledge in clinical guidelines.

In this work, we aimed to develop a semantic information model that could effectively characterize the details of disease phenotypes for clinical guidelines. A semantic information model named PhenoSSU (semantic structured unit of phenotype) was developed based on the clinical guidelines for 193 infectious diseases from Wikipedia. A total of 12 attributes were included in PhenoSSU, which characterized the details of phenotypes from various aspects. Based on PhenoSSU, we constructed fine-grained phenotype knowledge graphs for these infectious diseases. Considering the increased annotation costs associated with the introduction of PhenoSSU, we also explored the potential of machine learning for performing automatic recognition for PhenoSSU based on free text. It is hoped that our work will contribute to the large-scale construction of fine-grained phenotype knowledge graphs for more diseases.

## Methods

### Materials

We collected the clinical guidelines for 193 infectious diseases from Wikipedia [15] as the corpus for constructing fine-grained phenotype knowledge graphs. In Wikipedia, the phenotypic knowledge of infectious diseases was usually buried in a section named signs and symptoms (Multimedia Appendix 1). Although Wikipedia is created and edited by volunteers worldwide, many studies have proven the high quality of its biomedical content [16,17]. In addition, phenotype knowledge graphs for WikiData [3] and DBpedia [5] were also constructed based on clinical guidelines from Wikipedia.

### Design of PhenoSSU

PhenoSSU, by its very nature, is an entity-attribute-value model that consists of a phenotype concept along with a collection of attributes. Determining the attributes associated with various phenotypes is the key to the design of PhenoSSU. Four inclusion criteria for attributes were considered in this study:

- Introduced attribute and value set should come from a standard medical ontology to avoid the arbitrariness of defining new attributes. Systematized Nomenclature of Medicine–Clinical Terms (SNOMED-CT) [18,19], one of the most comprehensive clinical terminology databases in the world, was selected as the standard for normalizing both phenotypes and attributes.
- Introduced attribute should be a modifier associated with phenotypes rather than an entity independent of phenotypes. The concepts found in SNOMED-CT were organized into 19 distinct hierarchies. Phenotypes and attributes were mainly located in the clinical finding and qualifier value hierarchies, respectively (Multimedia Appendix 1).
- Value set of the introduced attribute should contain categorical variables with limited dimensionality. For example, the severity attribute in SNOMED-CT contains a value set including mild, moderate, and severe. This criterion is for convenience when configuring attributes in the brat rapid annotation tool (BRAT) [20] (Multimedia Appendix 1).
- Introduced attribute should occur at least once in the studied corpus. This criterion is for reducing redundancy when introducing many unused attributes.

**Figure 1:**
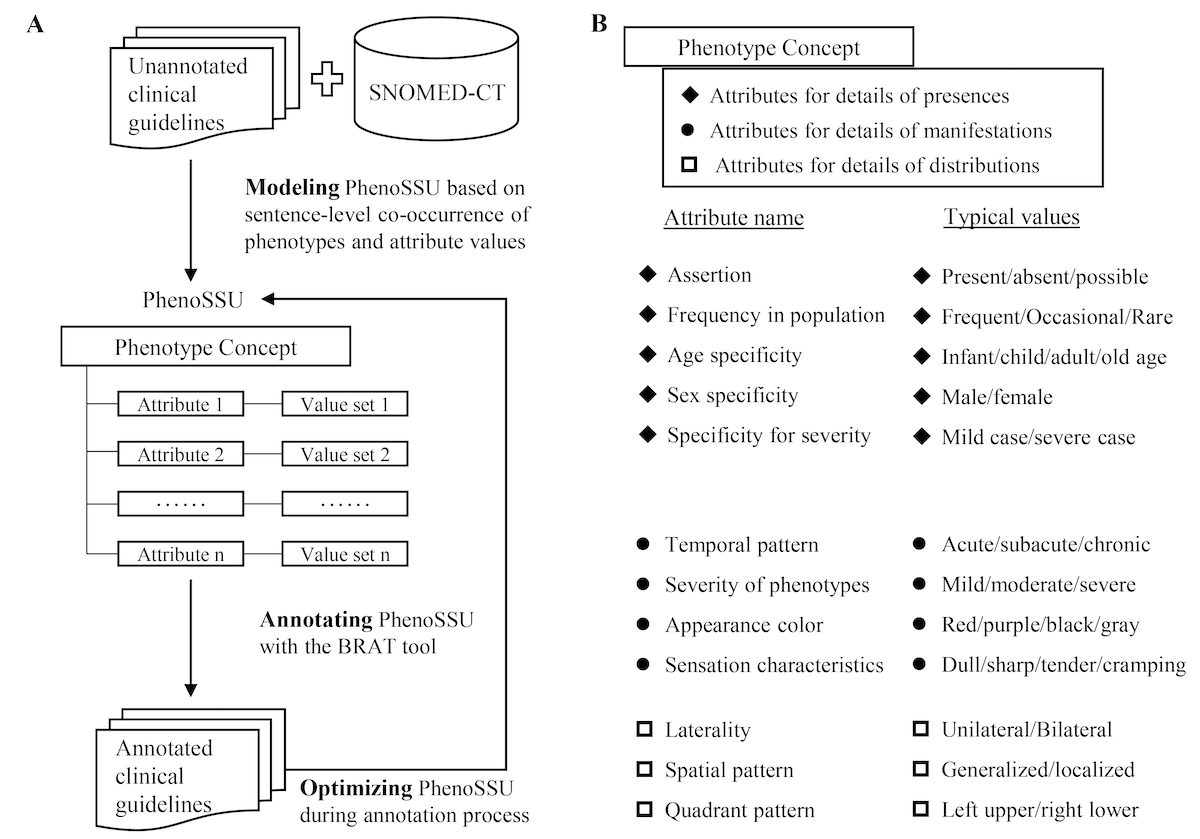
Modeling process of PhenoSSU: (A) modeling PhenoSSU based on sentence-level cooccurrences of phenotype concepts and attribute values in clinical guidelines and (B) components of the PhenoSSU model consist of a phenotype concept and 12 attributes.

To effectively find the attributes associated with various phenotypes, we developed a simple co-occurrence–based method for attribute filtering (Figure 1A). Specifically, the phenotypes in the corpus were annotated with the MetaMap tool [21], a state-of-the-art concept recognizer, and the values of the attributes in the corpus were annotated with the Flashtext tool [22], a string-based concept recognizer. If an attribute co-occurred with any phenotypes in at least 2 sentences from the whole corpus, we selected the attribute as a candidate that was potentially associated with phenotypes. Then, we manually filtered the attributes that were truly related to phenotypes and built an initial version of PhenoSSU. The initial PhenoSSU model was optimized during the annotation process. When annotators found a new contextual property associated with phenotypes, we searched for its existence in SNOMED-CT and added the standard attribute corresponding to that contextual property into the initial PhenoSSU model.

**Figure 2:**
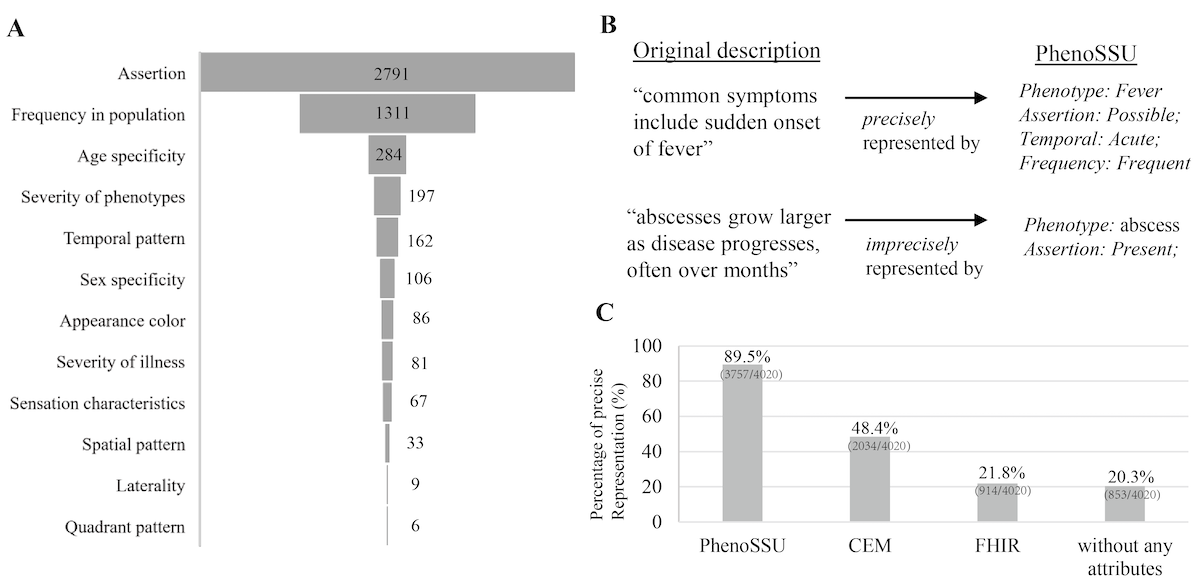
Expressive power of PhenoSSU: (A) prevalence of the 12 attributes in the studied corpus, (B) examples of precise and imprecise representations for original phenotype descriptions with the PhenoSSU model, and (C) comparisons of precise representation percentages among different information models.

The final PhenoSSU model contained 12 attributes, which could be classified into 3 categories according to the phenotypic details they characterized (Figure 1B): (1) details about the presence of phenotypes, including a phenotype’s assertion, frequency in a population, age specificity, sex specificity, and specificity regarding the severity of illness; (2) details about the manifestations of phenotypes, including a phenotype’s temporal pattern, severity, appearance color, and sensation characteristics; and (3) details about the spatial distributions of phenotypes, including a phenotype’s laterality, spatial pattern and quadrant pattern. The SNOMED-CT codes, definitions, and value sets of these attributes are listed in Multimedia Appendix 1. The distribution of these 12 attributes in the studied corpus is shown in Figure 2A.

### Annotation and Normalization of PhenoSSU

The annotation task of PhenoSSU can be divided into 2 steps: annotating a phenotype and annotating the attributes associated with that phenotype. Some annotation examples of different phenotypes attributes defined in PhenoSSU are presented in Multimedia Appendix 1. The clinical guides of 193 infectious diseases were annotated with the BRAT (Multimedia Appendix 1). To facilitate the annotation process, we preannotated the phenotypes found in clinical guidelines with the MetaMap tool. Then, two annotators (TY and SL) independently annotated the 193 clinical guidelines by following the annotation guide developed by LD and TJ. Their independent annotations were merged and visualized in the BRAT. To mark inconsistent annotations, we introduced a virtual attribute named agreement into PhenoSSU. Two independently annotated PhenoSSU models were regarded as consistent when both their phenotypes (text spans) and associated attribute values were the same. If there were inconsistencies in any part of a PhenoSSU model, the value of the agreement attribute was set to disagreement. The initial interannotator agreement at the PhenoSSU level was calculated with a Cohen kappa statistic [23] of 0.861. All inconsistent annotations were solved by an adjudication process (TJ).

The phenotypes annotated in BRAT were normalized with SNOMED-CT. To facilitate the normalization process, we also leveraged the MetaMap tool to obtain candidate concepts from the SNOMED-CT database and then manually selected the concept corresponding to each query phenotype. There was no need to normalize the attribute values because they were already normalized in SNOMED-CT.

One aspect to note about the normalization process is the special treatment used for finding sites of phenotypes. Finding sites were not explicitly included in the PhenoSSU model because they are entities independent of phenotypes. In SNOMED-CT, there were more than 39,000 concepts of finding sites in the body structure hierarchy, and these were hard to set as a value list in the BRAT. However, finding sites are indispensable information for describing phenotypes. Therefore, we also annotated the entities of finding sites associated with phenotypes. Taking the annotation of “bleeding from the nose and gum” as an example, the entities of the phenotype (bleeding) and two finding sites (nose, gum) were annotated separately and connected with a relation curve named locate (Multimedia Appendix 1). If a phenotype had an associated finding site, the phenotype together with the finding site was regarded as an integral concept in the normalization process. For example, the annotation of “bleeding” associated with “nose” was normalized as “249366005|epistaxis,” which shared the same codes as the annotation of “bleeding from nose.” If a composite concept could not be normalized as a whole (eg, “rash associated with hands”), we standardized the phenotype and its corresponding finding site separately and combined them into a postcoordination expression [24] (eg, “271807003|Rash”: “33712006|Skin structure of hand”; Multimedia Appendix 1). In summary, information about finding sites was implicitly considered an integral part of a phenotype concept rather than its attribute.

### Automatic Recognition of PhenoSSU

The manual annotation of a PhenoSSU model is a very time-consuming process because annotators not only need to find the mention of a phenotype but also need to determine the existence of attribute trigger terms in the context surrounding a phenotype. To reduce annotation costs, it is necessary to develop algorithms for the automatic annotation of PhenoSSU models.

The recognition task of PhenoSSU can be divided into 2 subtasks: phenotype concept recognition and attribute value prediction. The first subtask aims to recognize the text spans corresponding to phenotypes, and the second subtask aims to select appropriate values for 12 attributes based on a phenotype’s context.

The 193 annotated clinical guides were randomly divided into a training set and a test set at a ratio of 6:4. For the subtask of phenotype concept recognition, we still used the MetaMap tool, which can recognize phenotype concepts based on the Metathesaurus in the Unified Medical Language System (2020AA release) [25]. We optimized the parameters of the MetaMap tool based on its performance on the task of recognizing phenotype concepts in the training set (Multimedia Appendix 1).

The subtask of attribute value prediction can be regarded as a classification problem, and two machine learning-based models were explored for this subtask. One model was based on a support vector machine (SVM), and the other model was based on a bidirectional long short-term memory (BiLSTM) neural network. For the value classification model of a specific attribute, the input was the encoded feature vectors of a phenotype’s context and the output was one of the normalized values for this attribute.

We chose an SVM for developing attribute value prediction models because SVM-based models have proven their efficiency in the 2010 Informatics for Integrating Biology & the Bedside/Veterans Affairs challenge [26] and SemEval-2015 Task 14 [27]. In the SVM-based model (Multimedia Appendix 1), the context of a phenotype was encoded with the existence of trigger terms (terms that indicated a normalized value [eg, “sudden onset” was the trigger term of the normalized value “acute”]) and their distances to the target phenotype [26,27]. The SVM-based model was developed by using the scikit-learn package (version 0.23.1) [28]. The parameters of the SVM-based model were optimized by using a grid search strategy [29] on the training set.

Inspired by recent methodology developments for the assertion status prediction task [30,31], we chose BiLSTM for developing attribute value prediction models. The referenced studies [30,31] showed that BiLSTM and attention mechanisms could achieve better performances than other approaches when classifying assertions of medical concepts. Since assertion status prediction belonged to the task of attribute value prediction, we transferred the attention-enhanced BiLSTM model to our study. In a given BiLSTM-based model (Multimedia Appendix 1), the context of a phenotype was first split into 3 segments, including the left context, the phenotype itself, and the right context, which were then encoded into a 3×768 vector with a pretrained language model named BERT (bidirectional encoder representation from transformers) [32-34]. Each BiLSTM-based model was developed by using the Keras package (version 2.3.1) [35], and the BERT encoding process was performed by using the bert-as-service package (version 1.10.0) [36]. Considering the very imbalanced distributions of attribute values in our dataset (Multimedia Appendix 1), we used the synthetic minority oversampling technique [37] from the imbalanced-learn package (version 0.7.0) [38] to balance the sample distributions. The hyperparameters of the constructed BiLSTM-based models were optimized using an early stopping strategy [39] on the training set.

### Evaluation of the Performance for Recognizing PhenoSSU

To evaluate the performance of the proposed algorithm in extracting PhenoSSU models from free text, we used the evaluation metrics from SemEval-2015 Task 14: Analysis of Clinical Text [27].

The evaluation metric for the subtask of phenotype concept recognition was the F1-score. A predicted phenotype concept was regarded as a true positive if its text span overlapped with a gold standard text span. The precision metric was calculated as the fraction of correctly predicted phenotypes among all phenotypes identified by MetaMap, and the recall metric was calculated as the fraction of correctly predicted phenotypes among all phenotypes identified by the annotators. The F1-score was calculated as the harmonic mean of precision and recall.

We chose the average weighted accuracy as the evaluation metric for the subtask of attribute value prediction because the distributions of different attribute values were very imbalanced. The average weighted accuracy metric considers the prevalence of an attribute value in the corpus, so it can measure how good an algorithm is at predicting the rare values of an attribute. The detailed calculating process of the average weighted accuracy can be found in Multimedia Appendix 1.

### Evaluation of the Expressive Power of PhenoSSU

Since the aim of this work was to develop a semantic information model that was more suitable than current approaches for representing phenotype knowledge in clinical guidelines, it was necessary to evaluate whether the annotated PhenoSSU model could capture the full semantics underlying the original descriptions of phenotypes. For example, in Figure 2B, the description “common symptoms include sudden onset of fever” could be perfectly represented by the PhenoSSU model (phenotype: fever; assertion: possible; frequency: frequent; temporal pattern: acute). By comparison, the description “abscesses grow larger as disease progress, often over months” was only partially represented by the PhenoSSU model (phenotype: abscess; assertion: present), which missed the information regarding the course and duration of abscess associated with the description.

To evaluate the expressive power of PhenoSSU, we introduced a virtual attribute named “equal to the original description” into the PhenoSSU model. If the annotated PhenoSSU did not capture the full semantics of an original description, we set the value of this attribute to “partial.” Two annotators (TY and SL) independently evaluated the expressive power of the annotated PhenoSSU model. The initial interannotator agreement as measured with Cohen kappa statistic was 0.903 (3631/4020). We reached a consensus for those inconsistent judgments by an adjudication process (TJ).

## Results

### Overview of the PhenoSSU Model and PhenoSSU-Based Knowledge Graphs

To characterize the details of phenotypes for clinical guidelines, a semantic information model named PhenoSSU was proposed. With the introduction of 12 attributes associated with various phenotypes, the obtained knowledge graphs based on PhenoSSU were more fine-grained than those based on phenotype concepts. In this work, 193 PhenoSSU-based knowledge graphs for infectious diseases were constructed. At the concept level, we annotated 4020 phenotypic terms, 3962 of which could be normalized with 1508 concepts in SNOMED-CT. At the attribute level, we annotated 5278 nondefault attribute values (“present” was the default attribute value for the assertion attribute, and “none” was the default attribute value for other attributes), which indicated the widespread presence of contextual properties for phenotypes in clinical guides. The most commonly used attributes included assertion, frequency in a population, age specificity, phenotype severity, and temporal pattern (Figure 2A).

Since the knowledge graphs in WikiData were also extracted from Wikipedia, we compared our knowledge graphs with those in WikiData at the concept level. WikiData built knowledge graphs for 66 of the 193 diseases, and these graphs included 354 phenotype concepts. Our annotations covered 297 of the 354 (83.9%) phenotypes from WikiData. For the uncovered phenotypes, we could not confirm their existence on the corresponding webpages of Wikipedia (including current and historical webpages). Most of these uncovered phenotypes may come from the manual additions of volunteers, who made use of sources other than Wikipedia (Multimedia Appendix 1).

### Expressive Power of PhenoSSU for Representing Phenotype Knowledge

To evaluate the expressive power of the PhenoSSU model quantitatively, we manually analyzed whether a PhenoSSU instance could capture the full semantics underlying the corresponding descriptions of phenotypes (Figure 2B).

In this study, we annotated 4020 PhenoSSU instances, 3757 of which (89.5%) were determined to precisely represent the original phenotype knowledge described by natural language (Figure 2C). If we only considered the presence and absence of phenotype concepts (concept-based representation), the percentage of precise representations decreased to 20.3% (853/4200). This result further suggested the necessity of introducing the attributes associated with phenotypes into the developed model. We also analyzed the expressive power of the CEM and FHIR models for phenotypes and found that their percentages of precise representations were 48.4% (2034/4200) and 21.8% (914/4200), respectively. Most of the attributes defined in the CEM and FHIR models were not used in clinical guidelines except for the severity and laterality of phenotypes. The CEM model achieved a higher expression power than that of the FHIR model because it considered the uncertainty of phenotypes (assertion: possible), which is a frequently used attribute in clinical guidelines. Please see Multimedia Appendix 1 for detailed comparisons between the attributes used in the PhenoSSU, CEM, and FHIR models.

### Potential for Increasing the Speed of PhenoSSU Model Annotation With Machine Learning

**Figure 3:**
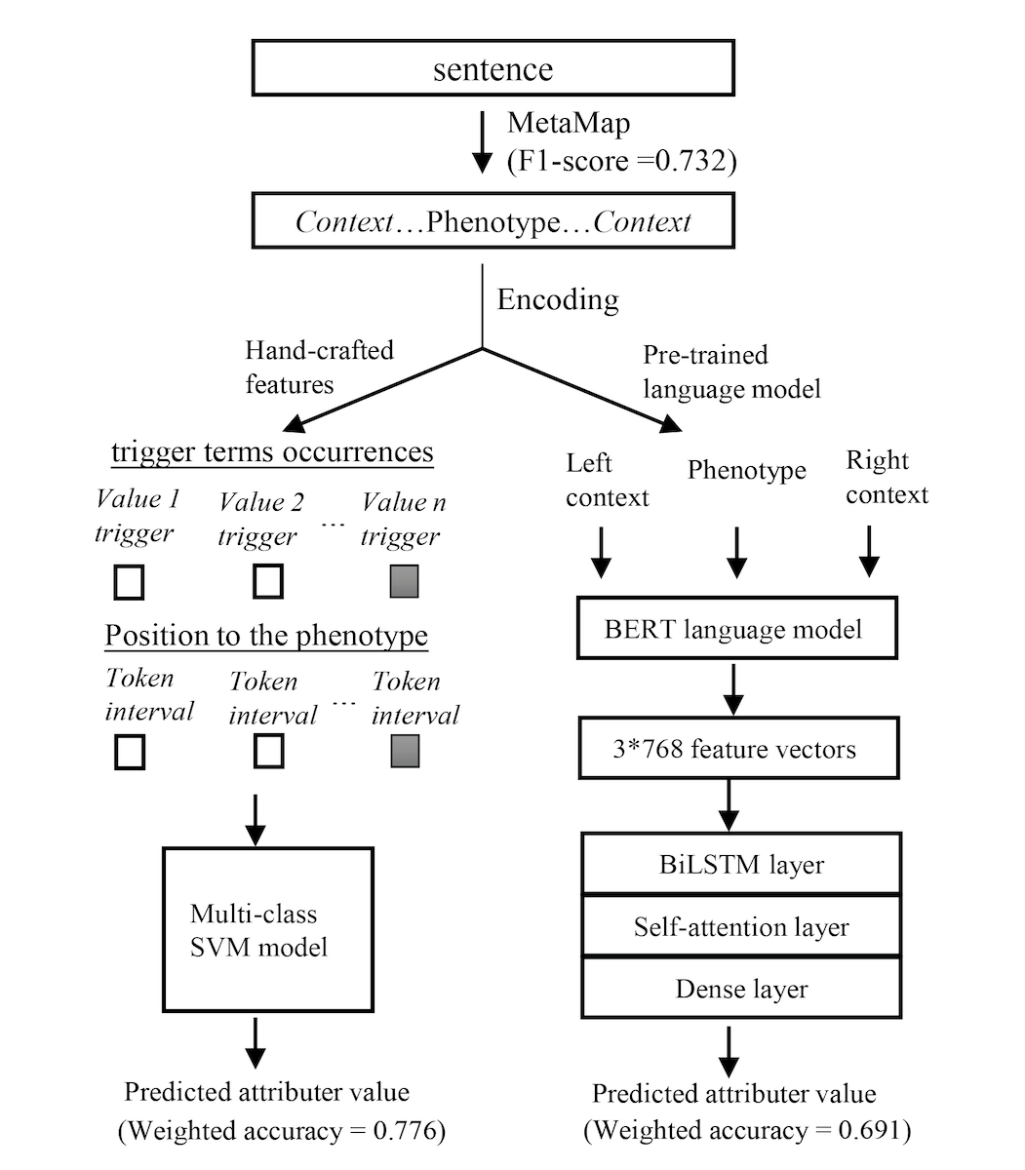
Automatic recognition of PhenoSSU.

With the introduction of attributes, it would take more time to annotate a PhenoSSU model than to annotate phenotype concepts. To increase the efficiency of annotating PhenoSSU models, we developed a hybrid strategy that first recognized phenotype concepts with the MetaMap tool and then predicted the attribute values of phenotypes with SVM-based or BiLSTM-based classifiers (Figure 3). For the subtask of phenotype concept recognition, the MetaMap tool achieved an F1-score of 0.732 (precision 0.660; recall 0.824), which was comparable to its performance on other medical corpora [40]. For the subtask of attribute value prediction, the average weighted accuracy of the SVM-based method (0.776) was better than that of the BiLSTM-based model (0.691). This may be due to limited number of training data, which made it hard for the deep learning-based approach to learn useful features from contexts. However, the performance of the BiLSTM-based model was still higher than the performance of a reference model (0.542) that always selected default values for attributes (it selected “present” for the assertion attribute and “none” for other attributes). These results indicate that machine learning methods have the potential to speed up PhenoSSU annotations. The detailed performances of the compared models for predicting the values of different attributes are listed in Multimedia Appendix 1.

## Discussion

### Principal Findings

In this work, we designed a fine-grained information model named PhenoSSU, which can precisely represent phenotype knowledge for clinical guidelines. We also developed an automatic strategy to extract PhenoSSU models from clinical guidelines and found that machine learning could be used to improve the efficiency of PhenoSSU annotation. Taken together, our work will provide a useful theoretical and technical guide for the construction of fine-grained phenotype knowledge graphs.

From the design of PhenoSSU, it can be seen that PhenoSSU was derived from SNOMED-CT because both the phenotype concepts and attribute values in PhenoSSU came from SNOMED-CT. PhenoSSU strengthened the expressive power of SNOMED-CT by combining 12 attributes with phenotype concepts. In SNOMED-CT, there was a technique named postcoordination expression [24] that could also capture the details of phenotypes by using combinations of existing concepts. For example, the out-of-vocabulary concept “severe headache, unilateral” can be expressed as a postcoordination of 3 concepts—headache (25064002): severity (272141005) = severe (24484000) and laterality (272741003) = unilateral (66459002). Compared with the postcoordination expression technique, PhenoSSU is a predefined information model that provides a general framework for knowledge representation. It is more convenient to configure the PhenoSSU model into the BRAT annotation tool to construct fine-grained phenotype knowledge graphs than to use the competing approach.

In recent years, machine learning, especially deep learning, has been widely used for processing medical information [41-44]. In this work, we also explored the potential of automatically constructing fine-grained phenotype knowledge graphs based on machine learning. The results in Figure 3 suggest that machine learning can assist with the human annotations of PhenoSSU to some extent. However, there are still great challenges to overcome to improve the performance of machine learning, especially the insufficiency and imbalanced distributions of training data. In future work, an active learning framework [45] that incorporates both human intelligence and machine intelligence may be a better strategy for constructing fine-grained knowledge graphs.

The improvement of knowledge granularity for disease phenotypes may potentially benefit knowledge-based diagnosis systems because the differential diagnostic capability of a PhenoSSU model is theoretically stronger than that of a single phenotype concept. From the perspective of coarse-grained knowledge graphs, some diseases (eg, the flu and common cold) have many similar symptoms (eg, fever and cough); however, these similar symptoms may have obvious differences from the perspectives of fine-grained knowledge graphs. For example, fever may be present in both flu and common cold. However, fever is more common in flu patients and usually appears suddenly with a body temperature of 38 degrees or above. By comparison, fever is rarely seen in common cold cases and usually appears gradually. Therefore, a diagnosis system cannot exclude the common cold if a patient has fever; however, it can safely exclude the common cold if a patient has such a PhenoSSU instance like “phenotype: fever; temporal pattern: acute; severity: severe.” PhenoSSU-based knowledge graphs should be very suitable for dialogue-based symptom checkers such as babylon [46] and symptoma [47], which collects the symptoms of a patient one by one. Considering the details of phenotypes in inquiry processing may potentially improve the efficiency and accuracy of dialogue-based symptom checkers.

### Limitations

One limitation of this work is that we only considered the corpus of infectious diseases during the modeling process of PhenoSSU. In addition, we only considered attributes with categorical values and did not consider attributes with numeric values. Another limitation of this study is that we only tested the effectiveness of the PhenoSSU model for 193 infectious diseases, which is a small number considering that thousands of other diseases exist. In addition, attributes suitable for infectious diseases may not be suitable for other types of diseases. We will solve these limitations during the process of constructing PhenoSSU-based knowledge graphs for more diseases in future work.

The annotation guidelines for PhenoSSU and the PhenoSSU-based knowledge graphs for 193 infectious diseases can be found by visiting our website [48]. The scripts for modeling and extracting PhenoSSU can be found on GitHub [49].

### Conclusions

PhenoSSU is a fine-grained semantic information model that can precisely represent phenotype knowledge in clinical guidelines, and machine learning can be used to improve the efficiency of constructing PhenoSSU-based knowledge graphs.

## Appendix

Multimedia Appendix 1 Supplementary figures, tables and texts.

## Abbreviations

BERT: bidirectional encoder representation from transformers
BiLSTM: bidirectional long short-term memory
BRAT: brat rapid annotation tool
CEM: clinical element model
FHIR: fast health care interoperability resource
PhenoSSU: semantic structured unit of phenotype
SNOMED-CT: Systematized Nomenclature of Medicine–Clinical Terms
SVM: support vector machine
