# Correction: Constructing High-Fidelity Phenotype Knowledge Graphs for Infectious Diseases With a Fine-Grained Semantic Information Model: Development and Usability Study

**DOI:** 10.2196/31481

**Published:** 2021-07-13

**Authors:** Lizong Deng, Luming Chen, Tao Yang, Mi Liu, Shicheng Li, Taijiao Jiang

**Affiliations:** 1 Center of Systems Medicine Institute of Basic Medical Sciences Chinese Academy of Medical Sciences & Peking Union Medical College Beijing China; 2 Suzhou Institute of Systems Medicine Suzhou China; 3 Jiangsu Institute of Clinical Immunology Jiangsu Key Laboratory of Clinical Immunology The First Affiliated Hospital of Soochow University Suzhou China; 4 Guangzhou Laboratory Guangzhou China

In “Constructing High-Fidelity Phenotype Knowledge Graphs for Infectious Diseases With a Fine-Grained Semantic Information Model: Development and Usability Study” (J Med Internet Res 2021;23(6):e26892) the authors noted three errors.

1. In the originally published manuscript, affiliation 1, 2, and 3 were incorrectly mentioned as follows: 

^1^Suzhou Institute of Systems Medicine, Suzhou, China

^2^Jiangsu Institute of Clinical Immunology, Jiangsu Key Laboratory of Clinical Immunology, The First Affiliated Hospital of Soochow University, Suzhou, China

^3^Center of Systems Medicine, Institute of Basic Medical Sciences, Chinese Academy of Medical Sciences & Peking Union Medical College, Beijing, China

These have been corrected to the following: 

^1^Center of Systems Medicine, Institute of Basic Medical Sciences, Chinese Academy of Medical Sciences & Peking Union Medical College, Beijing, China

^2^Suzhou Institute of Systems Medicine, Suzhou, China

^3^Jiangsu Institute of Clinical Immunology, Jiangsu Key Laboratory of Clinical Immunology, The First Affiliated Hospital of Soochow University, Suzhou, China

2. The affiliation *"Center of Systems Medicine, Institute of Basic Medical Sciences, Chinese Academy of Medical Sciences & Peking Union Medical College, Beijing, China"* was originally listed only for author *Taijiao Jiang*. This affiliation has now been added to authors *Lizong Deng, Luming Chen, Tao Yang,* and *Shicheng Li* in addition to author *Taijiao Jiang*.

3. The address of the corresponding author was originally published as follows:

Chongwen Road 100

Suzhou Industrial Park

Suzhou, 215000

China

This has been corrected to the following:

#5 Dong Dan San Tiao

Dongcheng District

Beijing, 100005

China

The correction will appear in the online version of the paper on the JMIR Publications website on July 13, 2021, together with the publication of this correction notice. Because this was made after submission to PubMed, PubMed Central, and other full-text repositories, the corrected article has also been resubmitted to those repositories.

