# Supplementary material for: Constructing High-Fidelity Phenotype Knowledge Graphs for Infectious Diseases With a Fine-Grained Semantic Information Model: Development and Usability Study
Source: J Med Internet Res. 2021 Jun 15;23(6):e26892. doi: 10.2196/26892 (PMC8277235; doi:10.2196/26892)
Supplement: Multimedia Appendix 1 [file jmir_v23i6e26892_app1.pdf]

Supplementary Materials

Menu

Supplementary Figures 2
Figure S1 2
Figure S2. 3
Figure S3. 3
Figure S4 4
Figure S5 4
Figure S6 5
Figure S7 5
Figure S8 6
Figure S9 6
Supplementary Tables 7
Table S1 7
Table S2 9
Table S3 10
Table S4 11
Table S5. 13
Table S6 14
Supplementary Texts 15
Text S1 15

Supplementary Figures

Figure S1. Concept-based representation for phenotypes of disease. Screenshots for phenotype knowledge graphs of influenza were taken from (A) WikiData and (B) MalaCards.

A

B

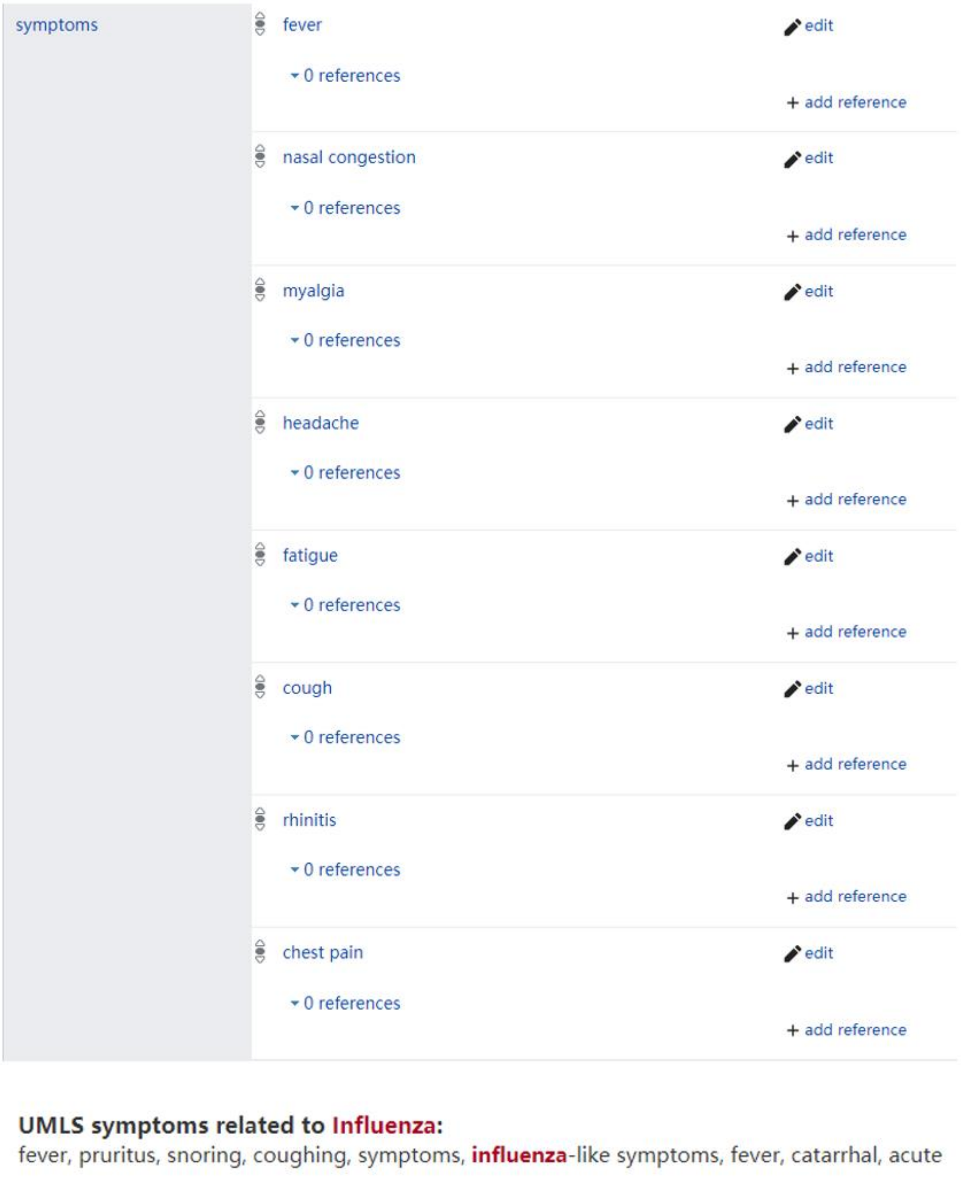

Figure S2. A screenshot of a clinical guideline from Wikipedia. In Wikipedia, phenotype knowledge of infectious diseases was usually buried in the section named "signs and symptoms".

Influenza, commonly known as "**the flu**", is an infectious disease caused by an influenza virus.[1] Symptoms can be mild to severe.[5] The most common symptoms include: high fever, runny nose, sore throat, muscle and joint pain, headache, coughing, and feeling tired.[1] These symptoms typically begin two days after exposure to the virus and most last less than a week.[1] The cough, however, may last for more than two weeks.[1] In children, there may be diarrhea and vomiting, but these are not common in adults.[6] Diarrhea and vomiting occur more commonly in gastroenteritis, which is an unrelated disease and sometimes inaccurately referred to as "stomach flu" or the "24-hour flu".[6] Complications of influenza may include viral pneumonia, secondary bacterial pneumonia, sinus infections, and worsening of previous health problems such as asthma or heart failure.[2][5]

Figure S3. A screenshot shows the 19 top hierarchies of SNOMED-CT. Phenotypes and attributes were mainly located in the clinical finding and qualifier value hierarchies, respectively

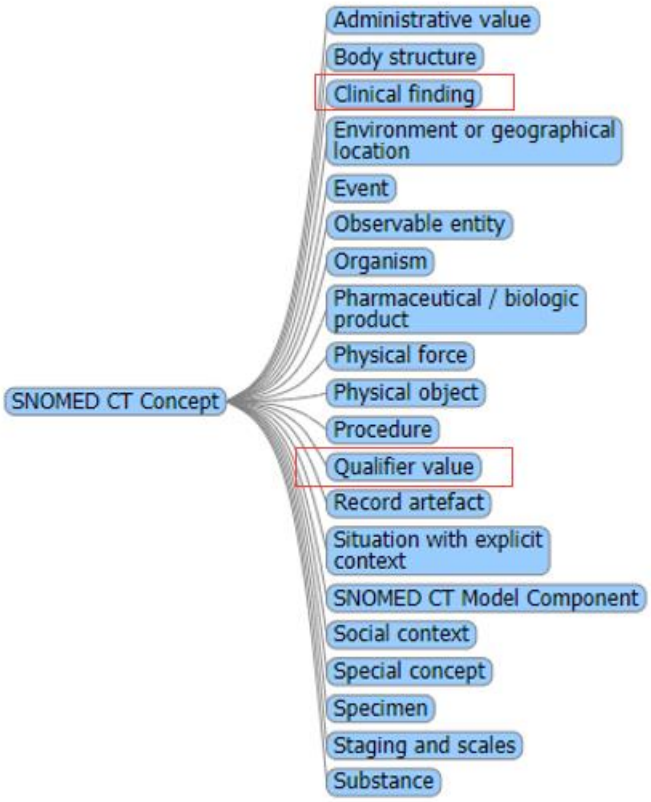

Figure S4. A screenshot shows configuring attributes of PhenoSSU into the BRAT annotation tool.

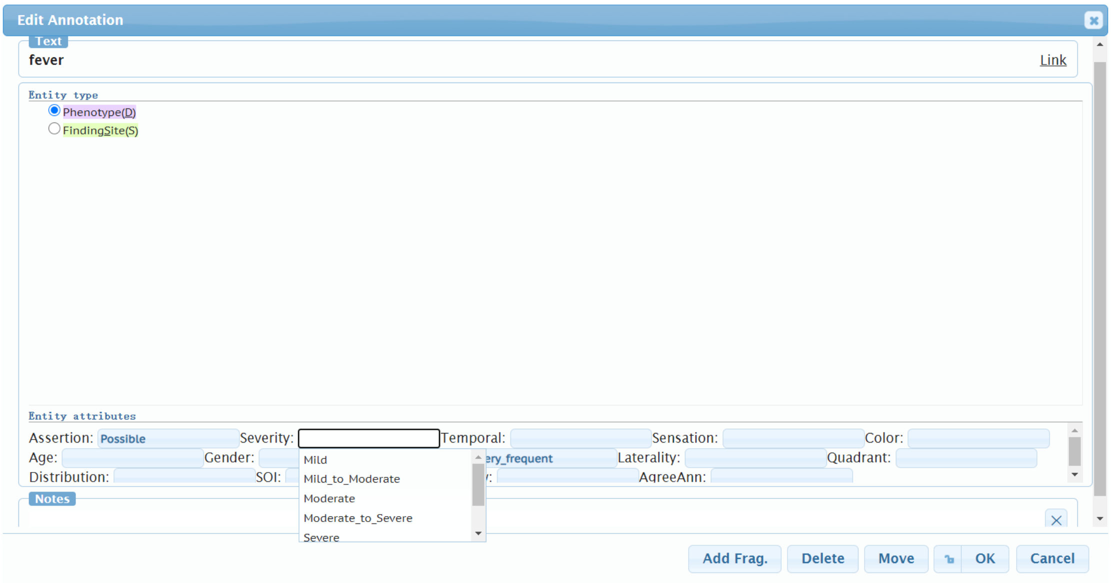

Figure S5. A screenshot shows an example of annotating PhenoSSU with the BRAT annotation tool.

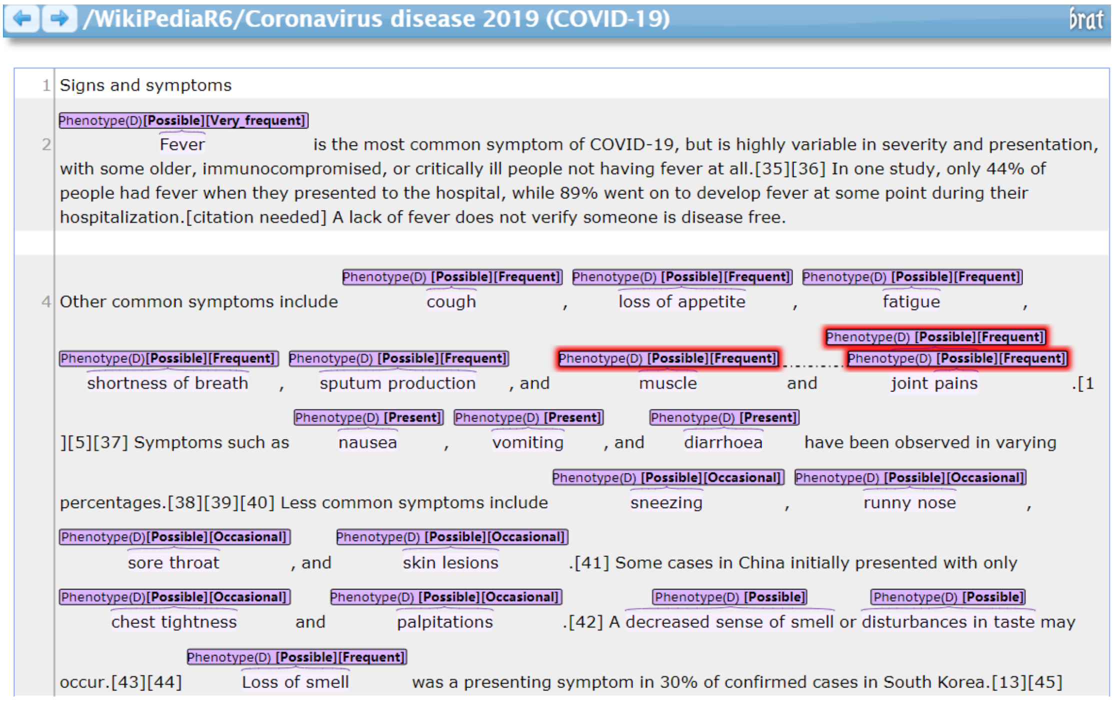

Figure S6. The normalization of phenotypes with finding sites by SNOMED-CT. (A) the phenotype "bleeding" and its associated finding site "nose" were treated as a composite concept to be normalized by SNOMED-CT; (B) A composite concept that cannot be normalized by SNOMED-CT were recorded in a post-coordination expression.

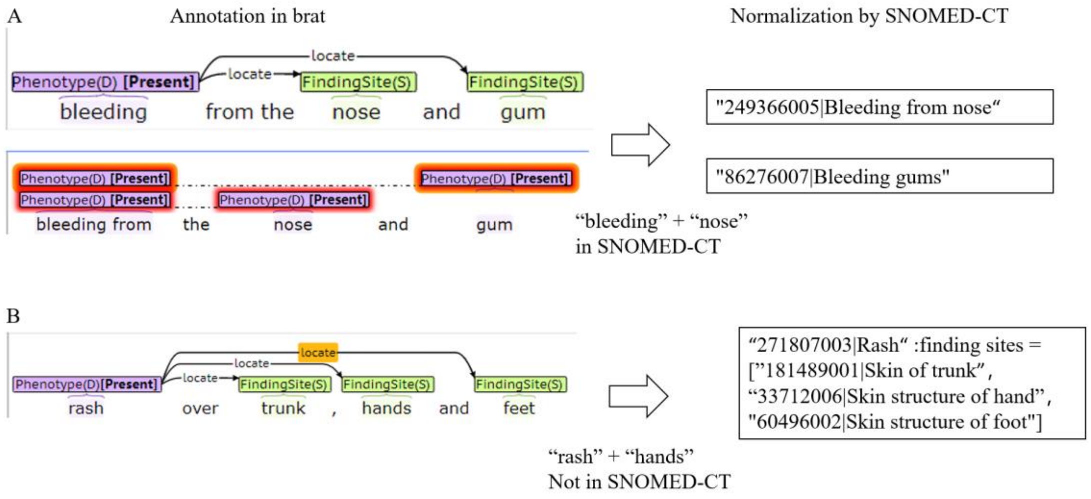

Figure S7. The architecture of SVM-based methods for predicting attribute value of a phenotype.

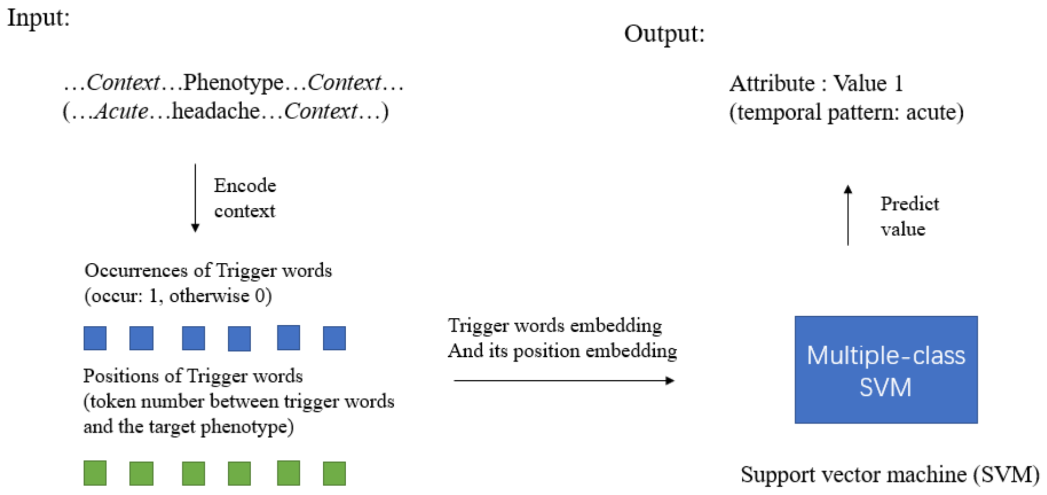

Figure S8. The architecture of BiLSTM-based methods for predicting attribute value of a phenotype.

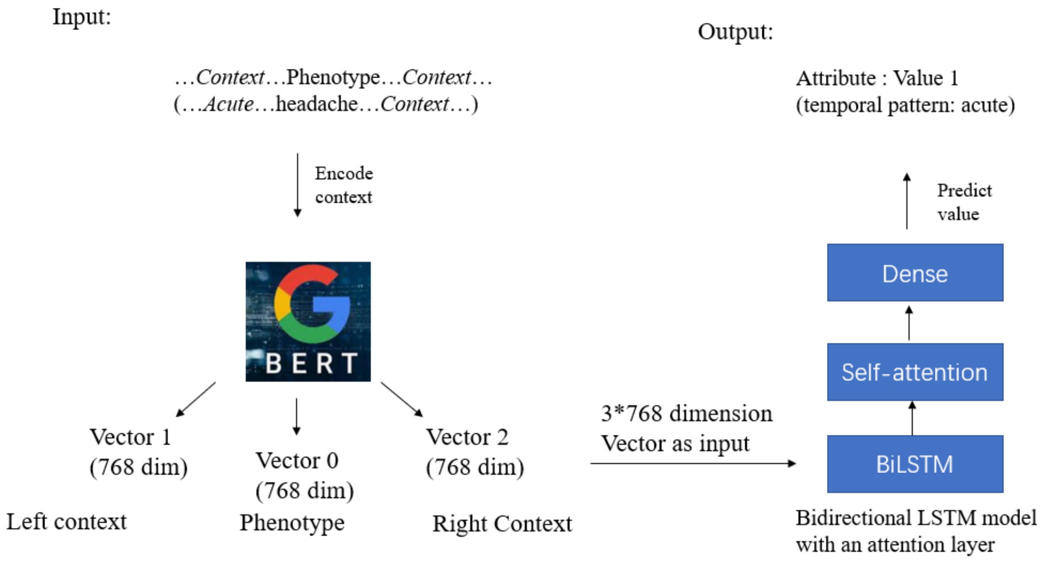

Figure S9. The screenshot for potential sources of phenotype terms in WikiData. There phenotype terms are not present in the original webpage of Wikipedia, but can be found in other sources.

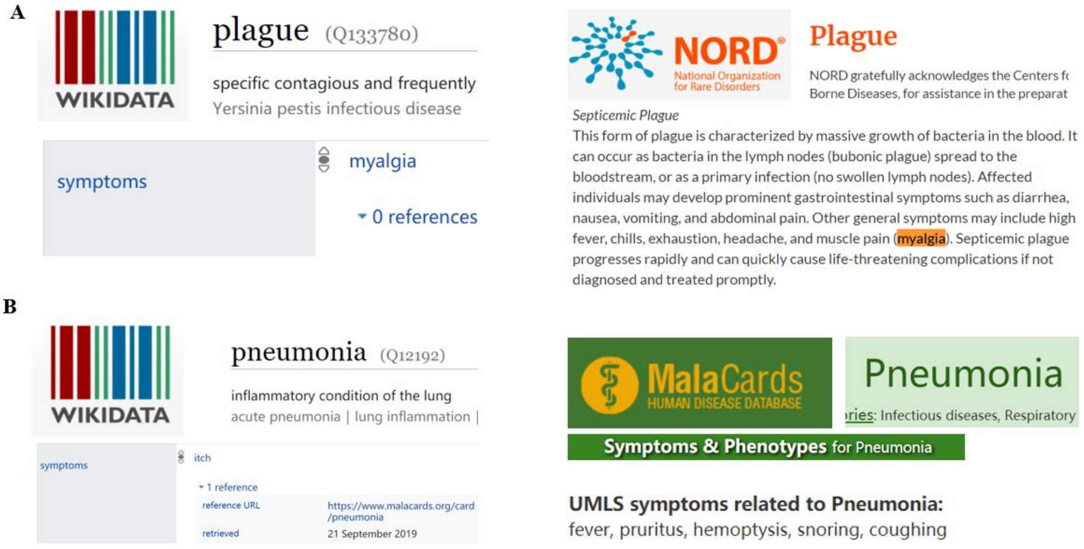

Supplementary Tables

Table S1. The definitions and value sets of attributes included in PhenoSSU. The SNOMED-CT code of an attribute value is shown in the form of "sct:code". Noted that only typical attribute values are listed if there are too many values for an attribute.

Attribute	Definition	Typical values
Assertion<br>(sct: 260245000)	a physician's belief status with regards to a particular patient's medical problem. The assertion is reflected in two aspects: whether the entity occurs to the patient, and how the entity occurs to the patient.	Present (sct:52101004);<br>Absent (sct:2667000);<br>Possible (sct:371930009);<br>Not associated with the patient (sct:255401001)
frequency in population<br>(sct: 272123002)	A contextual property to represent the frequency of phenotypic abnormalities within a patient cohort	Very frequent (99-80%) (sct:27732004);<br>Frequent (79-30%) (sct:70232002);<br>Occasional (29-5%) (sct:84638005);<br>Very rare (4-1%) (sct:89292003);
Age specificity<br>(sct: 282032007)	A contextual property to represent the specificity of phenotypic abnormalities in different periods in life	Antenatal (sct:263675000);<br>Neonatal (sct: 255407002);<br>Infancy (sct: 3658006);<br>Childhood (sct: 255398004);<br>Adolescent (sct: 263659003);<br>Adult (sct: 41847000);<br>Old age (sct: 271872005);<br>Pregnancy (sct:255409004);<br>Postpartum (sct: 255410009);
Sex specificity<br>(sct: 429019009)	A contextual property to represent the specificity of phenotypic abnormalities in male or female patients.	Male (sct: 248153007);<br>Female (sct: 248152002)
Specificity for severity of illness<br>(sct: 43749003)	A contextual property to represent the specificity of phenotypic abnormalities in mild or severe cases.	Mild case (sct: 162468002);<br>Severe case (sct: 162470006)
Temporal pattern<br>(sct: 272103003)	The speed at which disease manifestations appear and develop.	Acute (sct: 272118002),<br>Subacute (sct: 19939008),<br>Chronic (sct: 90734009),<br>Diurnal (sct: 28017001),

		Nocturnal (sct: 2546009),<br>Transient (sct: 14803004),<br>Prolonged (sct:255224006),<br>Recurrent (sct: 255227004),<br>Periodic (sct: 81591007),<br>Episodic (sct: 278499009)
Severity of phenotypes<br>(sct: 272141005)	The intensity or degree of a manifestation.	Mild (sct: 255604002);<br>Mild to moderate (sct: 371923003);<br>Moderate (sct: 6736007);<br>Moderate to Severe (sct: 371924009);<br>Severe (sct: 24484000);<br>Life threatening severity (sct: 442452003);<br>(body temperature:<br>37.1-38℃, mild;<br>38.1-39℃, moderate;<br>>=39.1℃ , severe)
Appearance color<br>(sct: 263714004)	The color of a manifestation.	Red (sct: 371240000),<br>Pink (sct: 371243003),<br>Yellow (sct: 263935005),<br>Yellow green (sct: 371248007),<br>Purple (sct: 371250004),<br>Black (sct: 371252007),<br>Gray (sct: 371253002),
Sensation characteristics<br>(sct: 272144002)	A subjective category or type of sensation.	Cold (sct: 84162001),<br>Hot (sct: 264018002),<br>Burning (sct: 62404004),<br>Painless (sct: 255350008),<br>Dull (sct: 263744001),<br>Sharp (sct: 410707003),<br>Tender (sct: 300820003),<br>Cramping (sct: 410709000)
Laterality<br>(sct: 272741003)	The localization with respect to the side of the body of the specified phenotypic abnormality.	Left (sct: 7771000),<br>Right (sct: 24028007),<br>Unilateral (sct: 66459002),<br>Bilateral (sct: 51440002)
Spatial pattern<br>(sct: 255464007)	The pattern by which a phenotype affects one or more regions of the body.	Generalized (sct: 60132005),<br>Localized (sct: 255471002),<br>Diffuse (sct: 19648000)<br>Focal (sct: 87017008)

Quadrant pattern<br>(sct: 272137006)	The pattern by which a phenotype affects the four quadrants of abdomen or breast.	Left upper quadrant (sct: 255481003),<br>Left lower quadrant (sct: 255480002),<br>Right upper quadrant (sct: 255497008),<br>Right lower quadrant (sct: 255495000)

Table S2. Examples of annotating different attributes of phenotypes defined in PhenoSSU.

Attribute	Value	Example
Assertion	Possible	people *may* have **vomiting** and **diarrhea**.
Frequency in population	Very frequent	*The most common* symptoms at onset of COVID-19 include **fever**, **cough**, and **shortness of breath**.
Age specificity	Childhood	In *children*, symptoms of **anorexia**, **nausea**, and **vomiting** have been investigated as possible symptoms.
Sex specificity	Female	In *women*, it causes **cervicitis** and **pelvic inflammatory diseases** (PID)
Specificity for severity of illness	Severe cases	In *severe cases*, persistent infections can lead to **enteropathy**, **intestinal villous atrophy**, and **malabsorption**.
Severity of phenotypes	Severe	Symptoms include *severe* **headache**, a sustained *high* **fever** …
Temporal pattern	Acute	Signs and symptoms begin with *sudden onset* of **fever**, and other flu-like symptoms about one to two weeks after being infected
Appearance color	Red	a small *red* **papule** (skin elevation) appears
Sensation characteristics	Sharp	shortness of breath, *sharp* or *stabbing* **chest pain** during deep breaths, and an increased rate of breathing
Laterality	Unilateral	Clinical signs include: *unilateral* upper motor neuron **limb weakness**, **cerebellar signs**, and **cranial nerve palsies**
Spatial pattern	Diffuse	The most common skin manifestation is a *diffuse* **erythematous rash, …**
Quadrant pattern	Right upper quadrant	pruritus, *right upper-quadrant* **abdominal tenderness** etc., are clinical manifestations indistinguishable from cholangitis

Table S3. Performances of MetaMap in recognizing phenotype concepts in the training set with different parameters. [1]Restrict to disorder group: semantic group of disorder in UMLS includes ['acab','anab','cgab','comd','dsyn','emod','fndg','inpo','mobd','patf','sosy'];

Parameters	Precision	Recall	F1 score
MetaMap;<br>[1]Restrict to disorder group = True;<br>Composite phrase = 2;<br>Ignore word order = True;<br>Allow concept gaps = True;<br>Word sense disambiguation=True	34.7%<br>(2783/8017)	93.6%<br>(2731/2917)	0.493
**MetaMapLite**;<br>[1]Restrict to disorder group = True;	33.2%<br>(2520/7573)	85.2%<br>(2486/2917)	0.478
MetaMap;<br>**Restrict to sources** =<br>**'SNOMEDCT_US'**;<br>[1]Restrict to disorder group = True;<br>Composite phrase = 2;<br>Ignore word order = True;<br>Allow concept gaps = True;<br>Word sense disambiguation=True	54.5%<br>(2370/4345)	84.2%<br>2459/2917	0.662
MetaMap;<br>**Restrict to sources** = [**'HPO'**];<br>[1]Restrict to disorder group = True;<br>Composite phrase = 2;<br>Ignore word order = True;<br>Allow concept gaps = True;<br>Word sense disambiguation=True	61.4%<br>(2261/3680)	77.7%<br>(2266/2917)	0.686
MetaMap;<br>**Restrict to sources** = [**'HPO'**];<br>[1]Restrict to disorder group = True;<br>**Composite phrase** = **0**;<br>Ignore word order = True;<br>Allow concept gaps = True;<br>Word sense disambiguation=True	61.2%<br>(2188/3536)	74.8%<br>2183/2917	0.673
MetaMap;<br>**Restrict to sources** = [**'HPO'**];<br>[1]Restrict to disorder group = True;<br>**Composite phrase** = **4**;<br>Ignore word order = True;<br>Allow concept gaps = True;<br>Word sense disambiguation=True	61.4%<br>2261/3680	77.7%<br>2266/2917	0.686
MetaMap;<br>**Restrict to sources** = [**'HPO'**];	67.4%<br>2166/3213	74.5%<br>2172/2917	**0.708**

[1]Restrict to disorder group = True;<br>**Composite phrase** = **0**;<br>**Ignore word order** = **False**;<br>**Allow concept gaps** = **False**;<br>Word sense disambiguation=True			

Table S4. The distribution of attribute values in the studied corpus.

Attribute values occurred in the corpus	Number of phenotypes with the value	Training set	Test set
Assertion = Present	1229 (30.57%)	862	367
Assertion = Possible	2692 (66.97%)	1976	716
Assertion = Conditional	75 (1.87%)	62	13
Assertion = Hypothetical	4 (0.10%)	4	0
Assertion = Absent	20 (0.50%)	13	7
Frequency in population = Obligate	2 (0.05%)	31	21
Frequency in population = Very frequent	166 (4.13%)	2	0
Frequency in population = Frequent	669 (16.64%)	8	1
Frequency in population = Occasional	309 (7.69%)	89	39
Frequency in population = Very rare	165 (4.10%)	5	1
Frequency in population = None	2709 (67.39%)	1932	777
Age specificity = Pregnancy	18 (0.45%)	14	4
Age specificity = Neonatal	9 (0.22%)	7	2
Age specificity = Infancy	58 (1.44%)	39	19
Age specificity = Childhood	112 (2.79%)	81	31
Age specificity = Adolescent	22 (0.55%)	22	0
Age specificity = Young adult	5 (0.12%)	5	0
Age specificity = Adult	49 (1.22%)	30	19
Age specificity = Old age	11 (0.27%)	11	0
Age specificity = None	3736 (92.94%)	2708	1028
Sex specificity = Female	70 (1.74%)	53	17
Sex specificity = Male	36 (0.90%)	21	15
Sex specificity = None	3914 (97.36%)	2843	1071
Severity of illness = Mild cases	17 (0.42%)	9	8
Severity of illness = Severe cases	64 (1.59%)	46	18
Severity of illness = None	3939 (97.99%)	2863	1077
Temporal pattern = Acute	70 (1.74%)	45	25
Temporal pattern = Subacute	10 (0.25%)	3	7
Temporal pattern = Chronic	46 (1.14%)	36	10
Temporal pattern = Recurrent	6 (0.15%)	4	2
Temporal pattern = Prolonged	14 (0.35%)	13	1

Temporal pattern = Transient	7 (0.17%)	6	1
Temporal pattern = Nocturnal	9 (0.22%)	6	3
Temporal pattern = None	3858 (95.97%)	2804	1054
Severity of phenotypes = Mild	52 (1.29%)	31	21
Severity of phenotypes = Mild to moderate	2 (0.05%)	2	0
Severity of phenotypes = Moderate	0 (0.00%)	0	0
Severity of phenotypes = Moderate to Severe	9 (0.22%)	8	1
Severity of phenotypes = Severe	128 (3.18%)	89	39
Severity of phenotypes = Life threatening	6 (0.15%)	5	1
Severity of phenotypes = None	3823 (95.10%)	2782	1041
Appearance color = Red	46 (1.14%)	33	13
Appearance color = Black	11 (0.27%)	6	5
Appearance color = Pink	4 (0.10%)	4	0
Appearance color = Purple	1 (0.02%)	0	1
Appearance color = Gray	6 (0.15%)	5	1
Appearance color = White	7 (0.17%)	4	3
Appearance color = Yellow	5 (0.12%)	3	2
Appearance color = Green	1 (0.02%)	1	0
Appearance color = Blue	3 (0.07%)	3	0
Appearance color = Brown	1 (0.02%)	0	1
Appearance color = Yellow green	1 (0.02%)	1	0
Appearance color = None	3934 (97.86%)	2857	1077
Sensation characteristics = Burning	15 (0.37%)	10	5
Sensation characteristics = Tender	19 (0.47%)	16	3
Sensation characteristics = Painless	12 (0.30%)	10	2
Sensation characteristics = Cramping	12 (0.30%)	8	4
Sensation characteristics = Hot	1 (0.02%)	1	0
Sensation characteristics = Tickling	1 (0.02%)	0	1
Sensation characteristics = Stabbing	2 (0.05%)	2	0
Sensation characteristics = Throbbing	1 (0.02%)	0	1
Sensation characteristics = Colicky	1 (0.02%)	1	0
Sensation characteristics = Pricking	2 (0.05%)	2	0
Sensation characteristics = Tinkling	1 (0.02%)	1	0
Sensation characteristics = None	3953 (98.33%)	2866	1087
Laterality = Left	0 (0.00%)	0	0
Laterality = Right	1 (0.02%)	1	0
Laterality = Unilateral	5 (0.12%)	5	0
Laterality = Bilateral	3 (0.07%)	2	1
Laterality = None	4011 (99.78%)	2909	1102
Spatial pattern = Generalized	17 (0.42%)	13	4
Spatial pattern = Localized	3 (0.07%)	3	0
Spatial pattern = Diffuse	8 (0.20%)	7	1
Spatial pattern = Focal	1 (0.02%)	1	0
Spatial pattern = Multifocal	3 (0.07%)	2	1

Spatial pattern = Distal<br>Spatial pattern = None	1 (0.02%)<br>3987 (99.18%)	1<br>2890	0<br>1097
Quadrant pattern = Left upper quadrant<br>Quadrant pattern = Left lower quadrant<br>Quadrant pattern = Right upper quadrant<br>Quadrant pattern = Right lower quadrant<br>Quadrant pattern = None	0 (0.00%)<br>0 (0.00%)<br>4 (0.10%)<br>2 (0.05%)<br>4014 (99.85%)	0<br>0<br>2<br>2<br>2713	0<br>0<br>2<br>0<br>1101

Table S5. Detailed comparations of attributes used in the PhenoSSU, CEM, and FHIR models. The information model for sign and symptom in CEM and the information model for condition in FHIR were used for comparison.

	PhenoSSU	CEM.symptom	FHIR.condition
Common attributes	● Severity of phenotypes<br>● Laterality<br>● Assertion (pesent/absent/not associated with patient/possible)	● Severity of phenotypes<br>● Laterality<br>● Negation indicator (absent)<br>● Uncertainty indicator (possible)<br>● Subject (not associated with patient)	● Severity of phenotypes<br>● Laterality<br>● Subject (not associated with patient)
Different attributes	● frequency in a population<br>● age specificity<br>● sex specificity<br>● specificity for severity of illness<br>● temporal pattern<br>● appearance color<br>● sensation characteristics<br>● spatial pattern<br>● quadrant pattern	● Alleviating/exacerbating factor<br>● Course<br>● Duration<br>● Start time<br>● End time<br>● Generic	● Clinical status<br>● verificationStatus<br>● encounter<br>● recordedDate<br>● recorder<br>● stage<br>● evidence<br>● Onset (onset age/datatime/period)

Table S6. The detailed performances for predicting values of different attributes on the test set.

Attribute	SVM-based model	BiLSTM-based model	Reference model (select default values)
Assertion	0.676	0.706	0.506
Severity of phenotypes	0.900	0.681	0.464
Temporal pattern	0.771	0.579	0.462
Sensation characteristics	0.895	0.754	0.550
Appearance color	0.794	0.636	0.407
Age specificity	0.823	0.644	0.472
Sex specificity	0.964	0.769	0.518
Frequency in population	0.769	0.610	0.454
Laterality	(Insufficient training data: sample <5)	(Insufficient training data: sample <5)	0.451
Quadrant pattern	(Insufficient training data: sample <5)	(Insufficient training data: sample <5)	0.404
Spatial pattern	0.909	0.757	0.595
Severity of illness	0.788	0.502	0.464
average weighted accuracy	0.776	0.691	0.542

Supplementary Texts

Text S1. The calculation of average weighted accuracy for evaluating the subtask of attribute value prediction.

We chose the average weighted accuracy (WA) as the evaluation metric for the subtask of attribute value prediction because the distributions of different attribute values were very imbalanced (**Table S4**). The average weighted accuracy metric considers the prevalence of an attribute value in the corpus, so it can measure how good an algorithm is at predicting the rare values of an attribute. Suppose that there were N phenotype concepts (C_i, i=1…N) and K attributes ($s_1 \dots s_k$) for each of the phenotypes (K=12 in this study). For an attribute of a specific phenotype, its gold-standard value and predicted value were denoted as gs_k and ps_k, respectively. To calculate the average weighted accuracy, we calculated the weighted accuracy of the model for predicting the attribute values of a given phenotype with the following formula:

$$per\_concept\_wa = \frac{\sum_{k=1}^{K} weight(gs_k) * I(gs_k, ps_k)}{\sum_{k=1}^{K} weight(gs_k)}$$

where I is the identification function; I(x, y) = 1 if x=y and 0 otherwise. For an attribute with M normalized values, the weight of an attribute value s_k^i was set as:

$$weight(s_k^i) = 1 - prevalence(s_k^i)$$

where the prevalence of value s_k^i was calculated as the percentage of s_k^i among all occurrences of attribute values s_k^i (i=1…M). This formula would give smaller weights to more prevalent values.

Based on per-concept weighted accuracy, the average weighted accuracy was calculated as:

$$WA = \frac{\sum_{i=1}^{N} per\_concept\_wa(C_i)}{N}$$

A correctly identified PhenoSSU model meant that both the phenotype concept and its attribute values were correctly predicted. For the overall evaluation of PhenoSSU recognition, we used the following formula:

$$Accuracy = \frac{\sum_{i=1}^{\#TP} per\_concept\_wa(TP_i)}{\#TP}$$

where TP (true positive) denotes correctly predicted phenotype concepts.
